# Healthcare utilization after mass trauma: a register-based study of consultations with primary care and mental health services in survivors of terrorism

**DOI:** 10.1186/s12888-022-04358-4

**Published:** 2022-11-18

**Authors:** Lise Eilin Stene, Siri Thoresen, Tore Wentzel-Larsen, Grete Dyb

**Affiliations:** 1 Norwegian Centre for Violence and Traumatic Stress Studies (NKVTS), Gullhaugveien 1-3, NO-0484 Oslo, Norway; 2 Department of Psychology, University of Oslo, Forskningsveien 3A, NO-0373 Oslo, Norway; 3 Centre for Child and Adolescent Mental Health, Eastern and Southern Norway, Gullhaugveien 1, NO-0484 Oslo, Norway; 4 Institute of Clinical Medicine, Faculty of Medicine, University of Oslo, Postboks 1171, Blindern, NO-0318 Oslo, Norway

**Keywords:** Health Services Research, Psychological Trauma, Mass Casualty Incidents, Disaster Planning, Stress, Psychological, Stress Disorders, Post-Traumatic, Terrorism, Crisis Intervention, Emergencies, Adolescent

## Abstract

**Background:**

Knowledge on healthcare utilization after mass trauma is needed to strengthen the public health preparedness to such incidents. Using register-based data, this study had a unique opportunity to investigate how young survivors’ use of primary care physicians (PCP) and mental health services (MHS) changed after a terrorist attack.

**Methods:**

We examined register-based data on PCP and MHS consultations among 255 survivors (52% male) of the 2011 Utøya youth camp attack in Norway 3 years before and after the attack, and their reason for encounter with the PCP according to the International Classification for Primary Care (ICPC− 2).

**Results:**

The PCP and MHS consultation rates (CR) were higher in female than male survivors both acutely and at long-term. The mean yearly CRs increased from 2.25 to 4.41 for PCP and 1.77 to 13.59 for MHS the year before and after the attack in female survivors, and from 1.45 to 3.65 for PCP and 1.02 to 11.77 for MHS in male survivors. The third year post-attack CRs for PCP were 3.55 and 2.00; and CRs for MHS were 5.24 and 2.30 in female and male survivors, respectively. Among female survivors, 76% consulted PCP and 12% MHS the year preceding the attack; post-attack 93% consulted PCP and 73% MHS the first year; decreasing to 87 and 40% the third year. Among male survivors, 61% consulted PCP and 7% MHS the year preceding the attack; post-attack 86% consulted PCP and 61% MHS the first year, and 67 and 31% the third year. As for PCP consultations, there was a particular increase in psychological reasons for encounter following the attack.

**Conclusions:**

This study indicates that it is important to anticipate an increased healthcare utilization several years following mass trauma, particularly of MHS. Both PCP and MHS practitioners played important roles in providing healthcare for psychological problems in young survivors of terrorism in a country with universal and largely publicly financed healthcare and a gatekeeping system. The healthcare utilization could be different in countries with other health systems or psychosocial care responses to mass trauma.

**Supplementary Information:**

The online version contains supplementary material available at 10.1186/s12888-022-04358-4.

## Background

Following traumatic experiences, increased physiological reactivity and persisting stress may contribute to a range of long-term mental and physical health problems, such as posttraumatic stress disorder (PTSD), depression, anxiety disorders, chronic pain and aggravation of pre-existing illness [1–9]. Young survivors may be particularly susceptible to longstanding adverse consequences, as posttraumatic health problems may interfere with their psychosocial development and education [10–13]. In order to prevent long-term illness, it is important to provide suitable and timely healthcare and psychosocial support to those in need. Yet, unmet health needs have been observed after mass trauma [14, 15]. International guidelines on the provision of psychosocial care after mass trauma have been developed largely based on a consensus of expert opinions [16–19]. It has been acknowledged that people may be affected heterogeneously and need different types of support. Therefore, it has been recommended to implement multilayered mental health and psychosocial support structures with an intervention pyramid where, for instance; providing practical help, a sense of security and strengthening the community and family supports are considered basic levels [20]. The next step is individual non-specialized mental healthcare from e.g. primary care practitioners. Finally, specialized mental health services should be provided if needed, e.g. in case of acute stress disorder, posttraumatic stress disorder or severe depression [21]. Yet, there is a scarcity of knowledge about the actual provision of health services to the survivors of terrorist attacks and other mass trauma [22]. The unpredictability of such incidents makes it difficult to develop methodologically sound studies on their potential impact on health and healthcare utilization. Existing research has major limitations. Most studies rely on cross-sectional assessments without the ability to study changes over time. Therefore, measurements at several time points are requested [23]. Furthermore, pre-trauma data have rarely been available, yet a baseline assessment is essential to estimate the true impact of an event. Moreover, prior research has often relied on self-reported information on health service utilization, which may be unspecific and prone to recall bias [24–27]. More detailed, accurate and objective data on affected individuals’ utilization of different types of health services are needed to strengthen the public health preparedness and response to terrorist attacks and similar events. Finally, there are few studies on youth, even though terrorist attacks and school shootings often affect adolescents and young adults [23]. This study responded to these shortcomings by using register-based data to investigate pre- and post-attack utilization of primary and secondary health services in survivors of the Utøya youth camp attack in Norway. We aimed to provide knowledge on the provision of primary and secondary healthcare in response to a terrorist attack. More specifically, our objectives were to investigate the female and male survivors’ consultations with primary care physicians (PCP) and secondary mental health services (MHS) before and after exposure to a terrorist attack and examine their reasons for encounter with PCP, with a particular focus on psychological reasons for encounter.

## Material and methods

### Setting

On 22 July 2011, a solitary right-wing terrorist committed two terrorist acts in Norway. First, he detonated a car bomb in the Oslo Government Quarter, killing eight individuals. Two hours later, he perpetrated a shooting spree at the summer camp of a political youth organization on the Utøya island. During the nearly 1.5 hour-long shooting, 69 individuals died. Most of them were teenagers. The Utøya attack is viewed as a severe trauma due to the scope of injuries and fatalities, its long duration, the young age of those concerned, and the fact that they were designated targets. Prior findings have revealed that the survivors were highly exposed to danger during the attack, and that nearly three of four of them experienced loss of close ones in the attack [3]. Hence, the survivors were considered to be at substantial risk of developing posttraumatic health problems. They were geographically dispersed after the attack and resided in rural and urban municipalities all over Norway. Many were also about to move away from their family home to start their studies. In order to provide psychosocial support and identify survivors at risk of developing health or social problems, a primary care based proactive outreach program was outlined [28]. It was recommended that municipal crisis teams contact all survivors directly after the attack. Next, that all survivors be appointed a designated contact person to ensure continuity in the follow-up and set up at least three screening assessments throughout the first year after approximately 6 weeks, 3 months, and 1 year [25, 26]. The intention was to use the lowest effective level of care with further referral to specialized health services or other relevant help services in case of high or persisting levels of symptoms or problems with daily functioning.

### Data collection and procedures

Altogether, 495 survivors who had been on the Utøya island during the shooting were identified through police records. Study invitations were sent by postal mail to 490 survivors; whereas four survivors aged < 13 years and one living abroad were excluded. Semi-structured face-to-face interviews were performed by trained health personnel at three waves, 4–5 months, 14–15 months and 31–32 months after the attack. We asked for consent to linkage to register-based data at wave 3. The study had an open cohort design at waves 1 and 2 where all the eligible survivors (*n =* 490) were invited to participate [29]. At wave 3 invitations were sent to the 355 (72%) survivors who participated at wave 1 or 2. Overall, 261 (53%) survivors participated at wave 3. Register-based data were unavailable for six survivors due to non-consent to linkage to register-based data or lack of personal identification number that is necessary for linkage. Hence, we collected register-based data from the 255 (52%) survivors who were included in this study.

### Ethics

Participants aged ≥16 years gave written informed consent. Parental consent was required before survivors aged < 16 years could participate in the study. The Norwegian Regional Committees for Medical and Health Research Ethics South East and North approved the study. The study procedures as well as characteristics of non-participation and loss to follow-up have been reported previously [29, 30].

### Measures

#### Register-based and administrative claims data

In this study we examined records from health registers and administrative databases for a total observation period of 6 years from 3 years before until 3 years after the attack (22.7.2008–21.7.2014). Every resident in Norway has a personal identification number (PIN) which makes it possible to carry out longitudinal research based on a combination of different registers. The PIN is recorded in encrypted form in the registers, and enables individual linkage of longitudinal health register data.

*Data on specialized health services* were collected from the Norwegian Patient Register (NPR). The NPR covers activity data from all Norwegian specialist healthcare institutions. It includes records of all consultations and admissions in Norwegian government-owned hospitals and outpatient clinics, as well as consultations with private contract specialists who receive government reimbursement. In order to mend a small percentage of missing data for the reimbursed private contract specialists in NPR [31], we verified these data with records on consultations with reimbursed private contract specialists from the Norwegian Health Economics Administration (HELFO) database. Reporting of data on individual patient care is mandatory and linked to the governmental reimbursement system for the health services. Data on individual patient care are available from January 1, 2008 [32]. In this study, we retrieved data on the survivors’ consultations within specialized child and adolescent psychiatric services and adult psychiatric services, which we together named mental health services (MHS). To calculate the MHS consultation rates, we divided the number of consultations within child and adolescent psychiatry and adult psychiatry by the time period examined. If a survivor was admitted to a psychiatric hospital, we counted the day of admittance as one consultation and censored the days the survivor was in the hospital. Admissions to psychiatric hospital represented less than 1 % of the consultations (62/7135 (0.9%)).

*Data on primary health services* were retrieved from The Norwegian Health Economics Administration (HELFO) database. Primary care physicians send electronic compensation claims for all patient contacts to HELFO, which is responsible for the reimbursement through a national health insurance scheme. A claim identifies the patient, the reason for encounter according to the International Classification of Primary Care (ICPC-2), the fee codes for different types of contacts and the specialty of the practitioner. Consultations with Primary Care Physicians (PCP) covered consultations with the survivors’ regular General Practitioners (GPs) or other physicians working in general practice and primary care emergency units, including out-of-hours services. Based on the reason for encounter, we grouped the survivors’ PCP consultations into four categories considered relevant in the context of the terror attack:

1. *Psychological:* All PCP consultations registered with an ICPC-2 code within chapter P “Psychological” reason for encounter.
2. *Injury:* Consultations registered with the injury-related reasons for encounter listed in Additional file 1: Appendix 1.
3. *Administration/assault-related:* PCP consultations with the ICPC-2 codes A97 “No disease”, A98 “Health maintenance/prevention” and Z25 “Assault/harmful event problem”. This category was created to assess if there could be a change in consultations with PCP after the attack due to screening assessments as part of the proactive outreach program where the PCP did not necessarily consider the survivor to have a particular diagnosis or disorder.
4. *Other:* Consultations with any other ICPC-2 code than those stated above.

For consultations with more than one ICPC-2 code, priority was given to the category «psychological», next “injury”, and then “administration/assault-related”.

Our measurement of consultations covered actual meetings with a PCP or MHS practitioner. It did not include telephone calls, mail correspondence or laboratory visits only to perform prescribed blood tests, injections or similar.

#### Other data

We examined differences between female and male survivors on a set of interview-based variables considered potentially relevant for the access to and/or need for health services; namely age, country origin, financial situation, living in a central or peripheral location, hospitalization after attack, exposure level, and psychiatric and somatic symptoms. Age was dichotomized according to the Norwegian age of minority into younger than 18 years and 18 years or older at the time of the attack. Furthermore, non-Norwegian origin was defined as having both parents born abroad. Previous research indicates that survivors with non-Norwegian origin more were overall less satisfied with the healthcare follow-up than survivors with Norwegian origin [30]. The survivors’ financial situation was assessed by a question on how they perceived their own (if they did not live with the parents) or their parents’ (if they lived with the parents) financial situation compared to others. We dichotomized the five response alternatives into financially disadvantaged (much or somewhat poorer) or not (similar, somewhat better, and much better). Survivors were defined as having a peripheral residence if their home municipality at the first study wave 4–5 months after the attacks, was located more than 45 minutes’ travelling time from communities with at least 15,000 inhabitants according to Statistics Norway’s classification of centrality [33]. Information about survivors who were hospitalized directly after the attack was verified with medical journals. Attack exposure was measured by a sum score of 13 potentially traumatic events occurring during the attack. It was assessed in the first study wave, except for survivors who first participated in the study at the second wave, for whom it was assessed 14–15 months after the attack. The exposure assessment was constructed to cover critical events experienced at the island during the attack, and has been described in details previously [3]. Posttraumatic stress symptoms (PTSS) in the past month were assessed by the University of California at Los Angeles Post-traumatic Stress Disorder (PTSD) Reaction Index (UCLA PTSD-RI) [34]. The total score includes 17 items conforming to the DSM–IV symptoms of PTSD rated on a five-point Likert scale from 0 (never) to 4 (most of the time) [35]. Three items have two alternative wordings, and are measured by the item with the highest score. Measures of PTSR were available for 235 survivors at wave 1 (4–5 months), 223 survivors at wave 2 (14–15 months), and 254 survivors at wave 3 (2.5 years after attack). There were 8 (3%) with 1 item missing in T1, 9 (4%) with 1 item missing in T2 and 6 (2%) with 1 item and 1 with 2 items missing in T3. The sum score was computed based on the mean of the valid items. Symptoms of depression and anxiety were evaluated with the mean score of the Hopkins Symptom Checklist-8 (HSCL-8), which is a short version of the HSCL-25. It measures symptoms of depression and anxiety the past 2 weeks with eight items scored from 1 (not bothered) to 4 (very bothered) [36]. Somatic symptoms during the past 2 weeks were assessed with a short version of the Children’s Somatic Symptoms Inventory (CSSI-8) [37]. The eight items covered pain in the stomach, head, lower back, and arms/legs; faintness/dizziness; rapid heartbeat; nausea/stomach problems; and weakness. The items were scored on a scale from 1 (not bothered) to 4 (very bothered). The short versions of the HSCL have displayed high psychometric qualities in population-based studies in Norway [38]. To our knowledge, there are no validation studies of the UCLA PTSD-RI or the CSSI-8 in the Norwegian population. A previous publication has documented that Cronbach’s alphas at the third study wave were 0.91 for the UCLA PTSD-RI, 0.90 for the HSCL-8, and 0.78 for the CSSI-8 [30]. At the third study wave 31–32 months after the attack, the survivors were also asked whether they, in relation to what they had experienced on Utøya, had paid for a private practicing psychiatrist or psychologist or another specialized medical doctor where they did not get all or part of the treatment covered by public sources.

### Statistics

**Fig. 1:**
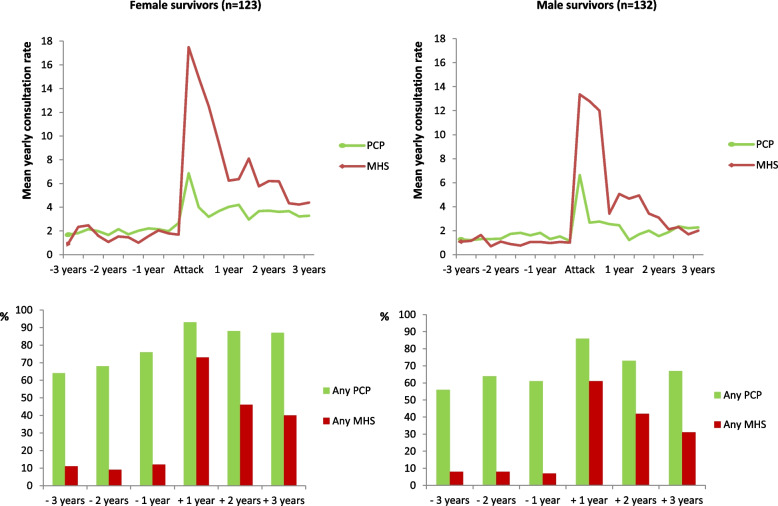
On top: The mean yearly consultation rates (CRs) for female (*n =* 123) and male (*n =* 132) survivors to primary care physicians (PCP) and secondary mental health services (MHS) three years before and after the Utøya attack. The mean yearly CRs have been calculated per quarter in the graphs in order to display changes in CRs in detail. Table 2 reports the actual mean CRs per year. Below: The percentage of female and male survivors with ≥1 consultations with primary care physicians (green columns) and secondary mental health services (red columns)

**Fig. 2:**
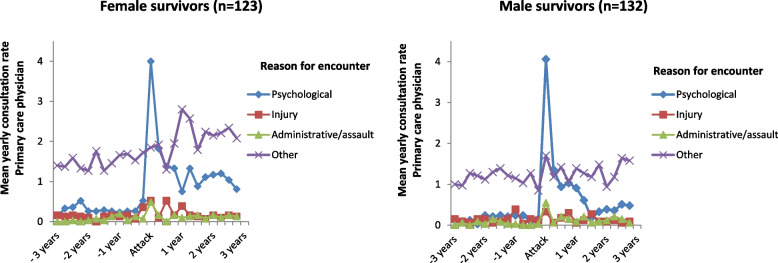
The mean yearly consultation rates with primary care physicians (PCP) by reasons for encounter (ICPC-2) before and after the Utøya attack 22.7.2011 for female (*n =* 123) and male (*n =* 132) survivors. The mean yearly CRs have been calculated per quarter in the graphs in order to display changes in CRs in detail. Table 3 reports the actual mean CRs per year in numbers

We calculated the mean yearly consultation rates (CRs) for female and male survivors in each of the 3 years preceding and following the attack (overall 6 years) by dividing the total number of consultations for each year by the person time in the study for the same year. Person time was estimated as total number of days in a specific year, and for those who were admitted to a psychiatric hospital, we censored (i.e. subtracted) the number of days in psychiatric hospital. We only censored days in psychiatric hospitals and not in somatic hospitals, because we observed that consultations with specialized mental health services also occurred during admittance at somatic hospitals. The graphs in Figs. 1 and 2 were based on mean yearly CRs calculated per quarter in order to display changes in CRs more detailed. The Tables 2 - 4 report the actual mean CRs per year in numbers. Furthermore, we computed 95% bootstrap BC_a_ confidence intervals (CI) for the differences in mean yearly CRs for MHS and PCP consultations comparing respectively the first, second and third year post-attack with the year preceding the attack through bootstrap computations with 10,000 replications. The results were presented separately for female and male survivors, since gender may influence the healthcare consultation frequency and reasons for seeking healthcare at baseline.

In order to compare characteristics of female and male survivors considered relevant for healthcare utilization, we used Pearson chi-squared tests for categorical variables and independent t-tests for continuous variables. We applied a two-sided level of significance at 0.05 for the *p*-value. The percentages and means were based on the total number of responses for each item. This study was based on survivors who participated in the third interview wave of a longitudinal study of the survivors of the Utøya attack [29]. We lacked data on symptoms at 4–5 months post-attack (wave 1) for 20 (7.8%) survivors and at 14–15 months post-attack (wave 2) for 32 (12.5%) survivors due to non-participation in those interviews. There were little missing data for those who participated in the respective interview waves: No respondents had more than two items with missing data in any of the symptoms scales (PTSD-RI, HSCL-8 and CSSI-8). If there was missing data on one or two items, we used the mean score of the answered items. Most analyses were conducted using IBM SPSS version 28, while the bootstrap computations used the R package boot.

## Results

Characteristics of female and male survivors are presented in Table 1. The mean age of the study participants at the time of the attack was 19.5 years. Female survivors were significantly more likely to be minors (< 18 years old) at the time of the attack, to have been severely injured with acute hospitalization directly after the attack and to have higher levels of posttraumatic stress symptoms, symptoms of depression and anxiety, and somatic symptoms in the early (4–5 months), intermediate (14–15 months) and long-term (31–32 months) aftermath of the attack.

**Table 1:** Characteristics of the 255 (52%) Utøya attack survivors in this study by gender

	Female (***n =*** 123)		Male (***n =*** 132)		
Characteristics of survivors	n/mean	(%/sd)	n/mean	(%/sd)	***p***-value
Minor at time of attack (< 18 y) (*n =* 255)					
Yes	63	(51.2)	51	(38.6)	0.043
No	60	(48.8)	81	(61.4)	
Non-Norwegian origin (*n =* 251)					
Yes	7	(5.8)	13	(9.9)	0.232
No	113	(94.2)	118	(91.1)	
Financially disadvantaged (*n =* 249)					
Yes	26	(21.5)	24	(18.8)	0.590
No	95	(78.5)	104	(81.2)	
Peripheral residence (*n =* 253)					
Yes	19	(15.6)	15	(11.5)	0.337
No	103	(84.4)	116	(88.5)	
Hospitalized directly after attack (*n =* 241)					
Yes	13	(10.6)	4	(3.0)	0.001
No	110	(89.4)	128	(97.0)	
Mean level of exposure, 0–13 (*n =* 252)	8,53	(2.22)	8,41	(2.10)	0.659
Posttraumatic stress symptoms					
(mean sum of PTSD-RI)					
Wave 1 (*n =* 235)	29,5	(11.9)	23.2	(11.6)	< 0.001
Wave 2 (*n =* 223)	23,8	(11.7)	18.6	(10.7)	< 0.001
Wave 3 (*n =* 254)	22,8	(12.3)	16.2	(11.6)	< 0.001
Depression and anxiety symptoms					
(mean HSCL-8)					
Wave 1 (*n =* 235)	2,23	(0.68)	1.94	(0.59)	< 0.001
Wave 2 (*n =* 223)	1,95	(0.69)	1.69	(0.59)	0.003
Wave 3 (*n =* 254)	1,91	(0.70)	1.56	(0.57)	< 0.001
Somatic symptoms					
(mean CSSI)					
Wave 1 (*n =* 235)	1,88	(0.55)	1.57	(0.50)	< 0.001
Wave 2 (*n =* 223)	1,81	(0.56)	1.50	(0.43)	< 0.001
Wave 3 (*n =* 254)	1,67	(0.51)	1.43	(0.41)	< 0.001

Altogether, 114 (93%) of female survivors and 116 (88%) of male survivors consulted PCP in the 3 years preceding the attack, compared to respectively 120 (98%) and 126 (96%) in the 3 years following the attack. Furthermore, 21 (17%) of female survivors and 15 (11%) of male survivors consulted MHS in the 3 years preceding the attack, compared to respectively 98 (80%) and 86 (65%) in the 3 years following the attack. Among the 36 survivors who had consulted MHS in the 3 years preceding the attack, 33 (92%) also consulted MHS in the 3 years following the attack. Overall 35 (14%) survivors reported that they had consulted psychiatrists, psychologists or another specialized medical doctor where they did not get all or part of the treatment covered by public sources. All these survivors had publicly funded MHS consultations recorded in the registry. Figure 1 displays the consultation rates (CR) and the percentages of female and male survivors who consulted PCP and MHS before and after the attack. The mean yearly CRs and percentages are presented in numbers in Table 2. As can be seen in Fig. 1 and Table 2, there was a clear increase in consultations both with PCP and MHS after the attack, yet the increase was most pronounced for MHS. Furthermore, Table 3 displays that the CRs after the attack were significantly higher in the three years following the attack, compared to the year preceding the attack, for both MHS and PCP consultations in both female and male survivors.

**Table 2:** Mean yearly consultation rates (CRs) and percentage of survivors with at least one consultation with primary care physicians (PCP) and specialized mental health services (MHS) by number of years before or after the Utøya attack 22.7.2011 among 123 (48%) female and 132 (52%) male survivors

Type of health services	Number of years before (−) or after (+) the Utøya attack					
	-3 years	-2 years	−1 year	+ 1 year	+ 2 years	+ 3 years
**Primary Care Physicians**						
*Mean yearly CRs*						
Female	1.91	1.89	2.25	4.41	3.72	3.55
Male	1.27	0.94	1.45	3.65	2.09	2.00
*Any consultations (*≥ *1), n (%)*						
Female	79 (64.2)	84 (68.3)	94 (76.4)	114 (92.7)	108 (87.8)	107 (87.0)
Male	74 (56.1)	85 (64.4)	80 (60.6)	114 (86.4)	96 (72.7)	88 (66.7)
**Mental Health Services**						
*Mean yearly CRs*						
Female	1.84	1.27	1.77	13.59	6.62	5.24
Male	1.14	0.95	1.02	11.77	4.52	2.30
*Any consultations (*≥ *1), n (%)*						
Female	13 (10.6)	11 (8.9)	15 (12.2)	90 (73.2)	56 (45.5)	49 (39.8)
Male	11 (8.3)	11 (8.3)	9 (6.8)	81 (61.4)	56 (42.4)	41 (31.1)

Mean yearly consultation rates (CRs) and percentage of survivors with at least one consultation with primary care physicians (PCP) and specialized mental health services (MHS) by number of years before or after the Utøya attack 22.7.2011 among 123 (48%) female and 132 (52%) male survivors.

**Table 3:** Increases in mean yearly consultation rates with primary care physicians (PCP) and specialized mental health services (MHS) the first, second and third year after the Utøya attack 22.7.2011 compared to the year preceding the attack in 123 (48%) female and 132 (52%) male survivors

	Increases in mean yearly consultation rates after the attack					
Type of health services	First year	95% CI	Second year	95% CI	Third year	95% CI
**Primary Care Physician**						
Female survivors	2.16	(1.55, 2.82)	1.46	(0.94, 2.02)	1.30	(0.72, 1.89)
Male survivors	2.20	(1.65, 2.82)	0.64	(0.19, 1.22)	0.55	(0.13, 1.03)
**Mental Health Services**						
Female survivors	11.82	(9.54, 14.72)	4.84	(3.35, 6.69)	3.46	(2.20, 5.14)
Male survivors	10.75	(8.36, 13.83)	3.50	(2.23, 5.04)	1.28	(0.28, 2.36)

Increases in mean yearly consultation rates with primary care physicians (PCP) and specialized mental health services (MHS) the first, second and third year after the Utøya attack 22.7.2011 compared to the year preceding the attack in 123 (48%) female and 132 (52%) male survivors. CI=Confidence interval.

Figure 2 illustrates the CR to PCP by categories of reasons for encounter, while Table 4 presents the numbers for the mean yearly CRs for the same categories. Following the attack, there was an early surge in PCP consultations with psychological reason for encounter (P-code in ICPC-2) both in female and male survivors.

**Table 4:** Mean yearly consultation rates (CR) with primary care physicians (PCP) by reasons for encounter (ICPC-2) according to number of years before or after the Utøya attack 22.7.2011 in 123 (48%) female and 132 (52%) male survivors

	Mean yearly CR with PCP by number **of years before (−) or after (+) the Utøya attack**					
**Reason for encounter (ICPC-2)**	−3 years	− 2 years	−1 year	+ 1 year	+ 2 years	+ 3 years
*Psychological*						
Female	0.33	0.27	0.32	2.13	1.02	1.06
Male	0.09	0.23	0.18	1.85	0.51	0.44
*Injury*						
Female	0.16	0.11	0.18	0.39	0.26	0.19
Male	0.11	0.11	0.17	0.23	0.15	0.09
*Administrative/assault-related*						
Female	0.01	0.07	0.11	0.25	0.11	0.14
Male	0.02	0.04	0.08	0.30	0.13	0.14
*Other*						
Female	1.42	1.46	1.71	2.11	2.55	2.45
Male	1.03	1.28	1.26	1.46	1.40	1.48

Mean yearly consultation rates (CR) with primary care physicians (PCP) by reasons for encounter (ICPC-2) according to number of years before or after the Utøya attack 22.7.2011 in 123 (48%) female and 132 (52%) male survivors.

Overall, there were 1162 PCP consultations with a psychological reason for encounter, 300 with injury-related reason for encounter (20 of these were additionally registered with and therefore classified as psychological reason for encounter in Fig. 2 and Table 4), 188 with administrative/assault-related reason for encounter (including 15 also registered with and classified as psychological reason for encounter and 2 registered with and classified as injury-related reason for encounter), and 2840 with other reasons for encounter (including 136 registered with and classified as psychological reason for encounter, 26 registered with and classified as injury-related, 27 registered with and classified as administrative/assault-related, and finally 2651 (93%) were classified as other reason for encounter). Table 5 reports the most frequent psychological reasons for encounter according to the ICPC-2 codes registered for the PCP consultations before and after the attack for female and male survivors, respectively.

**Table 5:** The overall most frequent psychological reasons for encounter (P-code in ICPC-2 classification) for consultations with primary care physicians in the 3 years before (pre-attack) and 3 years after (post-attack) the Utøya attack 22.7.2011 among 123 female and 132 male survivors

Most frequent psychological reasons for encounters with primary care physicians					
**Female survivors (*n =*** **123)**			**Male survivors (*n =*** **132)**		
Reason for encounter (ICPC-2)	No. of consultations		Reason for encounter (ICPC-2)	No. of consultations	
	Pre-attack	Post-attack		Pre-attack	Post-attack
P82 PTSD	< 5	180	P02 Acute stress disorder	5	123
P02 Acute stress disorder	7	147	P82 PTSD	< 5	115
P76 Depressive disorder	57	98	P29 Psychological symptom/complaint	15	35
P06 Sleep disturbance	< 5	29	P06 Sleep disturbance	< 5	28
P29 Psychological symptom/complaint	15	27	P76 Depressive disorder	6	17

The overall most frequent psychological reasons for encounter (P-code in ICPC-2 classification) for consultations with primary care physicians in the 3 years before (pre-attack) and 3 years after (post-attack) the Utøya attack 22.7.2011 among 123 female and 132 male survivors.

## Discussion

This study demonstrated that both PCP and MHS practitioners played important roles in providing healthcare for psychological problems in the young survivors of a terrorist attack in Norway. The majority consulted PCP both before and after the attack, with a clear increase in psychological reasons for encounter after the attack. Few of the survivors consulted MHS before the attack, while most of them did after the attack. In general, the consultations rates for PCP and MHS were higher for female than male survivors, both before and after the attack. In accordance with findings in previous research, the levels of posttraumatic stress symptoms, depression and anxiety symptoms and somatic symptoms were also higher in female survivors both early and at long-term after the attack [39, 40]. Hence, the higher consultation rates in female survivors might reflect higher needs for care. There was a major surge in the consultation rates both in female and male survivors the first year after the attack. The CRs declined the next 2 years, but remained elevated compared to the years before the attack.

Our findings demonstrated a high need for primary care and specialized mental health services several years following the terrorist attacks. These findings are in line with previous studies, indicating that the needs for care may persist for years after trauma exposure [41, 42] It is important to take this into account in the planning of the psychosocial care response. Furthermore, one should bear in mind that the use of health services may not correspond to the actual need for care. A prior study based on self-reports from the survivors of the Utøya attack conducted approximately 2.5 years after the attack, suggested that around 20% of the survivors scored their healthcare needs for psychological reactions due to the attack as higher than what they received [30]. It was nevertheless uncertain whether potential unmet needs were due to a lack of care or if the respondents received care they considered being unsatisfactory. The sample of our study was a severely exposed group of young survivors who were identified early after the attack. The healthcare needs and utilization may therefore have been particularly high. Since the attack occurred on a small island, it was possible to early identify all those who were directly exposed. In terrorist attacks occurring in places without distinct spatial/geographical limitations, survivors who are not seriously injured may evade the attack site and remain unidentified. In such settings, it may be more challenging to proactively offer psychosocial care, and access to care may depend more on self-referral.

In Norway, there is universal health coverage and a gatekeeping system with a regular GP scheme [43]. Our results indicated that there was a particular increase in the number of PCP consultations with a psychological reason for encounter after the attack. In the Norwegian health system, PCPs are both important providers of psychosocial care and gatekeepers for further referral to specialized MHS in order for the fees to be covered [43]. Consequently, a PCP consultation registered with a psychological reason for encounter may have represented mental healthcare provided by the PCP or a consultation for further referral to specialized MHS. It may also have been a consultation to get prescriptions for psychotropic medications or sick leaves due to mental health problems, or a combination of all these reasons. Hence, the survivors who did not receive specialized mental health services, may still have received basic mental healthcare from the PCPs or other primary care services. A prior study of the utilization of different types of health services in parents of the survivors of the Utøya attack, indicated that there was an increased contact with PCPs after the attack both for mothers and fathers [44]. For MHS, there was a significant increase in the contact frequency only among mothers, and not in the same scale as for the survivors. It may thus be that the parents’ health problems after the attack to a larger extent were handled at the primary care level.

Approximately nine of ten survivors had consulted PCPs in the 3 years preceding the attacks. We do not know whether these PCPs were the survivors’ regular GP, but one of the intentions of the regular GP scheme is that each inhabitant should have a regular GP to strengthen the continuity of care. Studies suggest that having a long-lasting patient relationship with a regular GP is associated with positive health-related outcomes [45, 46]. This may also be valuable to ensure good post-disaster follow-up, as regular GPs may have valuable knowledge about the survivors’ previous health problems and social situation, and may be important in the coordination and continuity of care throughout and beyond follow-up within MHS. Still, a study of a GP-based follow-up of Scandinavian survivors of the 2004 East Asian Tsunami catastrophe indicated that it may be challenging to organize a proactive post-disaster follow-up through the regular GPs [47].

The utilization of PCP and MHS after terrorist attacks could be different in countries with other health systems and/or different psychosocial care responses to terrorist attacks [48]. For instance, studies based on self-reported healthcare utilization suggest that the utilization of GPs was less common in survivors of the 13 November attacks in Paris, France than in survivors of the Utøya attack [24–26]. In France, psychosocial care the first month after terrorist attacks is provided by emergency psychosocial units mainly composed of specialized mental health personnel such as psychiatrists, psychologists and psychiatric nurses [49]. Furthermore, there is a different compensation system where patients may consult specialized mental health services directly and still get some, yet a smaller percentage, of the fees covered than when referred from a regular GP or other regular physician [50]. These factors may also impact the pattern of healthcare utilization after terrorist attacks. Examples of other countries than Norway that have a gatekeeping system with regular GP schemes, include the UK, the Netherlands and Denmark [51].

When we looked at openly available data on PCP consultations in the general population in Norway in the similar age spans 16–19 and 20–29 years, we observed that the consultations rates were generally higher for girls and women than men, which was also observed in our study [52]. Furthermore, these population data indicated that a very slight increase over time might be expected due to both increased age of the sample and a general tendency of increased use of PCP. This increase was also observed for PCP consultations with psychological reasons for encounter in the general population [53]. Nevertheless, the increases observed in the survivors after the terrorist attack were much larger than the slight increases over time observed in the general population.

### Strengths and limitations

This study provided detailed and continuous data on young survivors’ utilization of different types of health services before and after exposure to a terrorist attack. Using register-based data, we obtained objective information on the frequency and timing of healthcare utilization, as well as the type of healthcare used. In contrast to studies based on self-reports, these data are more specific and not prone to recall bias. Another strength of this study was the sample consisting of approximately half female and male survivors of young age exposed to the same severe and potentially traumatic event. The fact that the study participants were exposed simultaneously made it possible to compare some healthcare utilization measures with openly available healthcare data from the general population in similar age groups in the same period [52, 53]. Several limitations also apply to this study. We could not assess the quality of the care provided or whether the healthcare utilization corresponded to the healthcare needs. A prior study has in fact indicated that there were survivors who reported receiving either less or more healthcare than they needed [30]. Moreover, we did not have register-based data on the utilization of private healthcare fully paid by the patient. Nevertheless, self-reported information from the survivors indicated that most of them never consulted private practitioners without reimbursement. Furthermore, our assessment of the survivors’ reason for encounter with primary care physicians relied on the physicians’ classification of the consultation. The survivors may have consulted a PCP for several and different reasons, and we do not know to what extent the PCP correctly and exhaustively coded reasons for encounter. The fact that the PCP and MHS practitioners were aware of the survivors’ exposure to the terrorist attacks, may also have increased the likelihood of setting a diagnosis of PTSD or other psychiatric diagnoses, and increased the likelihood of referral to MHS. This study included 52% of the survivors of the Utøya attack who participated in the third study wave, and biases may have occurred. Prior research has demonstrated that there were no statistically significant differences between participants and non-participants in the Utøya study according to gender, age, hospitalization after the attack or peripheral residence [29, 30]. Survivors who were lost to follow-up after the two first study waves were significantly more often of non-Norwegian origin than participants in the third study wave, but they did not significantly differ with respect to levels of posttraumatic stress reactions or anxiety/depression symptoms [13]. However, participation in the study may have resulted in referrals to health services if unmet needs were detected during the interviews. This could potentially have increased the likelihood of long-term health service utilization among participants compared to non-participants. On the other hand, survivors with high levels of symptoms and health service utilization may have been less likely to participate in the study. Hence, potential biases could have caused an under- or overestimation of the health service utilization in this study.

### Clinical implications

Our findings indicate that the health services should anticipate and prepare for a large increase in the healthcare needs in young survivors of mass trauma. The increase is likely to be particularly high the first year, yet remain elevated several years compared to before the trauma exposure. Furthermore, the increase may be especially high for MHS, but also PCPs should expect a marked increase in consultations with psychological reasons for encounter, in particular related to posttraumatic stress symptoms, depression and sleep disorders (Table 5). Recent research has demonstrated that psychosocial care responses to mass trauma differ across countries, and that the long-term needs for follow-up are sometimes scarcely addressed in the plans for psychosocial care [48]. This study highlights the importance of planning a follow-up after mass trauma that efficiently meets both the survivors’ acute and long-term needs for healthcare. Following the Utøya attack, the Norwegian health authorities recommended a proactive psychosocial follow-up of the survivors lasting throughout at least the first year after the attack. The proactive follow-up was to be implemented by a contact person within the survivors’ local municipalities. However, a prior study indicated that only 55% of the survivors were followed up by the contact person throughout the entire year after the attack [26]. Moreover, a qualitative analysis found that many survivors experienced that the follow-up ended too soon [54]. Hence, it is essential to not only plan but also ensure the implementation of a follow-up that meets the needs for healthcare in the long-term as well as the acute phase.

### Future research

In order to enlarge the knowledge base on healthcare provision after terrorist attacks and other mass casualty incidents, accurate pre- and post-event data on healthcare utilization from registers or administrative databases should be analyzed also in future studies of trauma-exposed populations. Another important step forward would be to link register-based healthcare data with self-reported data from affected individuals on e.g. their psychological and somatic symptoms, their experiences and satisfaction with healthcare, and socioeconomic factors. This could yield a more profound insight into the relationship between healthcare utilization and symptom trajectories, whether there were unmet needs and potential predictors for unmet needs. Detailed data on utilization of specialized somatic health services would also be relevant to examine.

## Conclusion

Following a terrorist attack in a country with universal health coverage and a gatekeeping system, both PCP and MHS practitioners played important roles in the provision of healthcare for psychological problems in young survivors. Most survivors consulted PCP both before and after the attack, with a marked post-attack increase in psychological reasons for encounter. The majority of the survivors consulted MHS after the attack, whereas few did before the attack. The healthcare utilization was elevated several years after the attack compared to before the attack, and increased most for MHS. It is therefore important to prepare for an increased healthcare utilization several years following mass trauma. This study was conducted in a country with universal and mostly publicly financed healthcare and a regular GP-based gatekeeping system. The healthcare utilization after mass trauma may be different in countries with other health systems or psychosocial care responses.

## Supplementary Information

**Additional file 1.**

## Data Availability

The datasets generated and analyzed during the current study are not publicly available because individual privacy could be compromised but are available from the corresponding author on reasonable request.
