# Supplementary material for: Healthcare utilization after mass trauma: a register-based study of consultations with primary care and mental health services in survivors of terrorism
Source: BMC Psychiatry. 2022 Nov 18;22:720. doi: 10.1186/s12888-022-04358-4 (PMC9675057; doi:10.1186/s12888-022-04358-4)
Supplement: Supplementary file 1 — Additional file 1. [file 12888_2022_4358_MOESM1_ESM.docx]

**Appendix 1**

The following reasons for encounter, according to the International Classification of Primary Care (ICPC-2), were classified as injury:

A80 “Trauma/Injury NOS”, A81 “Multiple trauma/injuries”, A82 “Secondary effect of trauma”, A88 “Adverse effect physical factor”, B76 “Ruptured spleen traumatic”, B77 “Injury blood/lymph/spleen other”, D80 “Injury digestive system other”, F75 “Contusion/hemorrhage eye”, F76 “Foreign body in eye”, F79 “Injury eye other”, H76 “Foreign body in ear”, H77 “Perforation ear drum”, H78 “Superficial injury of ear”, H79 “Ear injury other”, L72 “Fracture: radius/ulna”, L73 “Fracture: tibia/fibula”, L74 “Fracture: hand/foot bone”, L75 “Fracture: Femur”, L76 “Fracture: other”, L77 “Sprain/strain of ankle”, L78 “Sprain/strain of knee”, L79 “Sprain/strain of joint”, L80 “Dislocation/subluxation”, L81 “Injury musculoskeletal NOS”, L96 “Acute internal damage knee”, N79 “Concussion”, N80 “Head injury other”, N81 “Injury nervous system other”, R87 “Foreign body nose/larynx/bronch”, R88 “Injury respiratory other”, S14 “Burn/scald”, S15 “Foreign body in skin”, S16 “Bruise/contusion”, S17 “Abrasion/scratch/blister”, S18 “Laceration/cut”, S19 “Skin injury other”, U80 “Injury urinary tract”, X82 “Injury genital female”, Y80 “Injury male genital”.
